# Challenge and Prospect of Traditional Chinese Medicine in Depression Treatment

**DOI:** 10.3389/fnins.2019.00190

**Published:** 2019-03-05

**Authors:** Yuan-Wei Zhang, Yung-Chi Cheng

**Affiliations:** ^1^School of Life Sciences, Guangzhou University, Guangzhou, China; ^2^Department of Pharmacology, School of Medicine, Yale University, New Haven, CT, United States

**Keywords:** depression, depression treatment, TCM, antidepressant, systems medicine, TCM formula

## Abstract

Current medication for depression is inadequate and far from ideal. Development of novel antidepressant drugs is a pressing task. The discovery of ketamine and related agents represents a new era in drug discovery for the rapid treatment of depression. Due to potential neurotoxicity, short-lasting efficacy, the limitation of a single target approach, and a limited role in depression prevention of these agents, additional approaches or drugs that exert synergy and compatibility, with the rapid-acting agents are required for better treatment of depression. Traditional Chinese Medicine (TCM) is a systems medicine and its clinical experience and integrated theory for diagnosis and treatment provides an alternative method of novel drug discovery in depression treatment. In TCM, there are numerous claimed effective antidepressant formulas, but comprehensive research and evidence-based clinical studies are required for their acceptance as a treatment. In this essay, we review current attempts in the discovery of new agents, TCM drug formulation, and TCM treatment of depression, and discuss the challenges and opportunities of TCM in the new era of antidepressant discovery. TCM could provide an important resource in the discovery of novel agents, assistance of the rapid-acting antidepressants, development of new agents for female patients, and the prevention of depression at its early stages. The study of depression in conjunction with TCM therefore not only provides an opportunity to scientifically evaluate the benefits and risks of TCM, but also accelerates the development of novel antidepressant agents by combining the principle of modern molecular medicine with the ideas of empirical systems medicine.

## Introduction

Depression is a chronic, recurrent, potentially life-threatening mental illness that affects approximately 15–20% of the global population (Manji et al., 2001; Charney, 2004) and it is thought to be caused by a complex interplay of genetic vulnerabilities and unfavorable environmental events. However, the specific genes or non-genetic events are poorly defined (Nestler et al., 2000). Many genes or physical and emotional factors might be involved in the etiology of depression. Most experts therefore agree that depression should be viewed as a multigenetic and multifactorial syndrome.

## Development of New Antidepressants

Current monoaminergic systems-based medications for the treatment of depression are far from ideal. Over the past three decades, although immense efforts have been focused on the development of novel treatments toward new potential targets beyond the monoamine hypothesis, none of these attempts have succeeded in the development of any fundamentally novel medications (Berton and Nestler, 2006).

The recent finding that ketamine, a NMDA receptor antagonist, produces rapid improvement in depression has shifted efforts toward novel agents that target the glutamatergic system (Berman et al., 2000; Zarate et al., 2006). The mechanism of ketamine action has been identified as the activation of AMPA receptors, which subsequently releases BDNF and activates mTOR signaling (Maeng et al., 2008; Zhou et al., 2014; Lepack et al., 2015). Studies on ketamine has led to the investigation of several rapid-acting agents that target the NMDA receptor, including the selective GluN2B antagonists, traxoprodil (Preskorn et al., 2008) and Ro-25-6891 (Li et al., 2011), and a NMDA receptor positive allosteric modulator, rapastinel (Burgdorf et al., 2013). Despite their drug abuse and neurotoxicity potential, these agents have shown promise as rapid-acting antidepressants and clinical studies are currently underway (Gerhard and Duman, 2018).

There are many reasons why searching for new antidepressants is difficult. First, most potential target proteins are broadly expressed throughout the brain and peripheral tissues and exert diverse physiological effects in different brain regions. It increases the concern of the neurotoxicity of any agent directed against these targets. Second, depression is a multifactorial and multigenetic syndrome, in which symptoms can vary from patient to patient. Depression is not a unified syndrome. It is impossible to find a specific gene responsible for induction or termination of depression in all patients. Third, single target approaches have been successful when applied to well-validated targets in the treatment of monogenic diseases, but is less effective for multifactorial and multigenic diseases, in which various entities with different underlying pathological mechanisms exist (Butcher, 2005). Therefore, improved strategies for effective treatment of depressive syndrome should simultaneously target dysregulation of several genes involved in the pathophysiology of depression. Moreover, considering their psychotomimetic, dissociative, and abuse potentials, the rapid-acting agents might be beneficial only for patients with severe forms of depression. For most patients with mild and moderate forms of depression, however, the best healing approach should be to identify the principal cause that is unique to each individual patient and then to apply an appropriate medical treatment or psychotherapy to correct the homeostatic imbalance. A systems biology-orientated healing approach to this problem may exist in Traditional Chinese Medicine (TCM).

## Tcm and Depression

Traditional Chinese Medicine is an experience-based medicine that has been developed in China over thousands of years, and emphasizes the integrity of the human body and the physical or emotional effects of the external environment on internal stability. Dynamic homeostasis is its basic principle. In TCM, all illnesses result from a homeostatic imbalance, which can occur because of a variety of social or environmental factors. Treatments therefore aim to restore internal balance. Based on its “systems” point of view, a typical treatment approach in TCM views the body as a whole entity and cures an illness not only by alleviating its symptoms, but also restoring internal balance.

**FIGURE 1:**
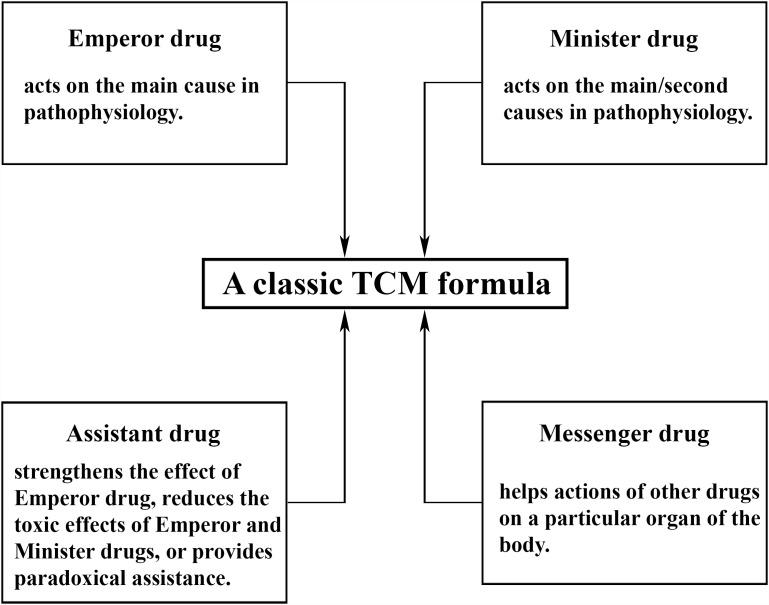
Composition of a typical Traditional Chinese Medicine (TCM) formula. In general, there are four classes of drugs in each formula: emperor drugs, minister drugs, messenger drugs, and assistant drugs. One TCM formula contains at least one emperor drug supplemented by minister, assistant, and messenger drugs at varying numbers from zero to multiple, in order to ensure effective actions on various targets simultaneously. The empirical composite formulas have been proven to have greater efficacy and safety than single drugs in clinical practices, possibly due to their synergistic interaction and mutual detoxification (Ung et al., 2007). The composition and dosage depend on the signs or symptoms of individual patients and can be modified to fit specific individuals more accurately, in accordance with the idea of individual therapy in modern medicine. The major risk of TCM is thought to be the use if a combination of several herbs with some uncertain factors, such as toxicity, consistency of bioactive ingredients, drug-drug interaction, and so on. All of these present major challenges the development of TCM but can be managed by proper processing to minimize toxic effects, standardization of formulas by quality control to ensure a consistent level of bioactive ingredients and reproducible pharmacological actions, proper dosing and avoiding usage at the same time as other drugs, respectively.

More importantly, in TCM, a combination of multiple drugs is often used to ensure the effective action on various targets (Figure 1). According to TCM, depression is caused by an imbalance within organ systems that eventually results in dysregulation of the brain function, and it is viewed as a multifactorial illness with a variety of pathological mechanisms in different patients. Depression may be caused by stagnation of “Qi” (vital energy), dysregulation of blood circulation, “Re” (inflammation), dampness and phlegm in the body. A TCM practitioner typically identifies the principal cause of depression that is unique to each patient and adopts an acupuncture or medical treatment. Release of stagnated vital energy is the general therapeutic principle for depression (Feng et al., 2016). It may be supplemented by activating blood circulation, suppressing inflammation, or by eliminating phlegm and dampness.

The systems, multi-drug, multi-target approach of TCM fits perfectly within the multifactorial pathophysiology of depression. There are numerous TCM empirical antidepressant formulas, which are widely used to treat patients with depression in East Asia today. Clinical research has demonstrated that these antidepressant formulas are effective (Yeung et al., 2015). On the other hand, although great efforts have been made to scientifically elucidate these formulas, our understanding of their mechanism of action, bioactive constituents, and pharmacology of synergy and compatibility of formulation, remains at a rudimentary level. When facing this challenge in the new era of antidepressant discovery, we wonder what can be learnt from ancient systems medicine in antidepressant discovery and how can empirical TCM be integrated into mainstream research, in the neuropharmacology of depression treatment? Here, we discuss future research directions and the possible role of TCM in the treatment of depression.

## Prospect of Tcm in Depression Treatment

Clinical experience and systems biology-orientated TCM provides an excellent paradigm to aid the development of novel medications for the effective pharmacotherapy of depression. In turn, the study of depression within TCM also provides an opportunity to scientifically interpret the benefits and risks of TCM antidepressant formulas.

The discovery of ketamine and related agents, represent a new era in drug discovery for the rapid treatment of depression. This does not mean that TCM cannot play a role in the development of novel antidepressants or approaches. Conversely, we expect that the study of TCM will attract attention and participation from outstanding scientists in the near future, for several reasons. First, side effects and the potential for drug abuse are critical problems these glutamate transmission-targeted rapid-acting agents face, although, it has been claimed that some agents have fewer psychotomimetic side effects than ketamine (Zarate et al., 2006; Preskorn et al., 2008). These side effects limit their chronic use. New agents that can be used on a daily sustained basis are required. Second, the rapid-acting agents are directed toward glutamate transmission and treatment with these agents increases spine synapse formation. However, new agent-induced synapses are lost after 1 week (Zarate et al., 2006; Abdallah et al., 2015). This reveals that glutamate transmission is a critical factor but not the principal cause in the pathophysiology of depression. Although these agents can rapidly but temporarily alleviate depressive symptoms, new agents that can sustain synaptic as well as the therapeutic actions of the rapid-acting agents are needed. Third, it is common that patients with depression also display other symptoms, such as chronic fatigue, chronic pain, heartburn, lupus, chronic constipation, and so on. Additional medications as adjuvants directed toward those symptoms are needed. Lastly, like monoamine-based antidepressants, rapid-acting agents have a limited value in preventing depression at its early stages. New approaches or agents that can correct internal imbalances in order to prevent early stage symptoms of depression are needed.

What can TCM do in the new era of new antidepressants discovery? Here, we list several aspects, not limited to, in which TCM can exert its excellence in the development of new antidepressants or approaches.

### Developing New Antidepressant Agents

Identification of bioactive ingredients will significantly improve our understanding of the mechanisms of action as well as benefits/risks of a TCM antidepressant formula at the molecular level. This effort will also be critical in developing a TCM antidepressant formula as a novel antidepressant agent. It can be exemplified with the study on ibogaine, a hallucinogenic alkaloid found in *Tabernanthe iboga*, and Yuanzhi-1, a triterpenoid saponin separated from *Polygala tenuifolia*. Both herbs are empirically used for the treatment of depression. Ibogaine has been shown to inhibit both serotonin and dopamine transporters but is unique among the transporter ligands in which it inhibits non-competitively and apparently binds to the extracellular surface of a cytoplasmic-facing transporter conformation (Jacobs et al., 2007; Bulling et al., 2012). On the other hand, Yuanzhi-1 was reported to inhibit all three monoamine transporters with a high potency in a low nanomolar range (Jin et al., 2014, 2015). The mechanism underlying antidepressant-like effects was thought to influence on monoaminergic systems, but lack of selectivity for serotonin or serotonin/norepinephrine reuptake increases concerns around their addictive side effects, due to rewarding effects evoked by elevated synaptic concentrations of dopamine in the brain.

Another example relevant to TCM is scopolamine, which is a natural compound isolated from the *Solanaceae* family of plants and which has been used for surgical anesthesia. Scopolamine is a non-selective mAChR antagonist and has been reported to have rapid antidepressant effects within days, through the blockade of mAChR1 on GABA interneurons (Drevets et al., 2013; Voleti et al., 2013; Wohleb et al., 2016a). These studies provide evidence that scopolamine treatment results in a rapid burst of glutamate in the medial prefrontal cortex and increases the number of spine synapses.

With many conventional and newly emerged pharmacological targets in depression treatment, including various neurotransmitter metabolic enzymes, transporters, receptors, second messenger systems, and proteins involved in the neurotrophic cascades, it should be feasible to identify bioactive constituents that exert specific interactions with the target proteins.

### Adjuvating the Rapid-Acting Antidepressant Agents

Although rapid-acting agents produce rapid antidepressant responses, the effects last for about 1 week, at which time patients typically relapse (Duman, 2018). Therefore, additional agents that have synergistic activity and compatibility with the rapid-acting agents are required to preserve therapeutic efficacy. In TCM practice, a patient with depression is usually given a formula to use on a daily sustained basis for several weeks, in order to alleviate depressive symptoms. The composite formula is not a unified formulation, but is rather based on symptoms within organ systems, prescribed to fit each individual patient and aims to activate blood circulation, eliminate phlegm and dampness, correct digestive and gastrointestinal dysfunction, or to improve immune function, etc. In this context, TCM could exert its strengths as an adjuvant to the rapid- acting antidepressants through different underlying mechanisms.

Among numerous TCM formulas, we used one prescribed for the treatment of gastrointestinal (GI) diseases, “Dai-Kenchu-To” (DKT in Japanese), as an example to further illustrate that TCM could be an ideal adjuvant. It is well known that the bidirectional communication between GI microbiota and the brain links the CNS with peripheral intestinal functions by means of neural, endocrine, immune, and humoral interactions (Rhee et al., 2009; Diaz Heijtz et al., 2011). Therefore, it has been suggested that the GI tract and microbiota are involved in the pathophysiology and etiology of depression (Schroeder et al., 2007; Desbonnet et al., 2008; Bravo et al., 2011; Arseneault-Breard et al., 2012). Patients with GI tract-induced depression might not show complete remission within a week after treatment with a rapid-acting antidepressant, as an additional medication, directed toward the GI tract, is also required for a better treatment.

Dai-Kenchu-To is a three-herb decoction used for the treatment of GI disorders, including postoperative paralytic ileus, ischemic intestinal disorders, irritable bowel syndrome, and Crohn’s disease (Hasebe et al., 2016). It has been reported that DKT treatment prevents bacterial translocation and maintains microbiome diversity in rats with acute stress (Yoshikawa et al., 2013). Studies have also showed that DKT increases intestinal blood flow in rats (Murata et al., 2002) and small intestinal movement in dogs (Jin et al., 2001), and prevents postoperative intestinal obstruction in rats (Tokita et al., 2007). From these studies, it is clear that DKT significantly improves GI tract function and can eliminate the risk of GI tract dysregulation in the pathophysiology of depression. Therefore, DKT should have a synergistic activity with the rapid-acting agents in GI tract-induced depression treatment, but additional research is required in order to assess its synergistic efficiency and compatibility.

### Developing New Antidepressant Agents for Women

Studies reported that females are twice as likely to suffer from depression (Kuehner, 2017) and that antidepressants have differential efficacy in males and females (Soldin and Mattison, 2009; Mazure and Jones, 2015). However, our knowledge on the gender-specific pathophysiology of depression is limited and currently no antidepressants specific to women are available in the market. It should be noted that women that suffer from depression present additional variables for the treatment of depression. Studies have revealed that women are more likely to experience depression during times of hormonal flux postpartum and during perimenopausal periods (Ahokas et al., 2001; Parker and Brotchie, 2004). Circulating sex hormones and differences in inflammatory, neurotrophic, and serotonergic responses to unfavorable events between sexes have been suggested as important factors in the pathophysiology of gender-specific depression (Bangasser and Valentino, 2014; Duman, 2017; Labonté et al., 2017). Therefore, a better understanding of the gender-specific pathophysiology of depression could help reveal novel and gender-specific therapeutic targets or approaches.

There are several TCM antidepressant formulas suitable for women which can be modified according to individual symptoms and additional variables. A representative formula for postpartum depression treatment is “Xiaoyao-san” (XYS), which is prepared from a mixture of eight herbs. It should be emphasized that in this formula, *Angelica sinensis* is commonly used to enrich blood, promote blood circulation, and to treat blood deficiency and menstrual disorders such as dysmenorrhea and irregular menstrual cycles (Wu and Hsieh, 2011). Preclinical studies have indicated that XYS exerts an antidepressant activity in stress-induced animal models through various underlying mechanisms, including the elevation of 5-HT contents in both the cerebral cortex and the hippocampus, the regulation of the HPA axis, the improvement of BDNF expression in the hippocampus, and the reduction of cytokine levels in serum (Jing et al., 2015). XYS has also shown immense promise as an antidepressant agent in postpartum depression treatment in several clinical studies (Li et al., 2016; Yang et al., 2018). However, the mechanism of action that underlies gender-specific depression treatment is poorly understood. Most of the preclinical studies on XYS have been performed in male rodents, and most clinical studies did not provide gender-specific effects of treatment responses. It is therefore necessary to conduct these studies in female animal models, to obtain gender-specific clinical data, particularly on the cardiovascular system, neuroendocrine responses, circulating hormones, and the cognitive control circuits, to improve our understanding of its mechanism of action.

### Preventing Depression at Its Early Stages

Clinical and basic studies have significantly improved our understanding of the pathophysiology of depression, revealing that numerous pathological factors are involved in the development of depression. Notable factors and systems include brain neurotransmitter systems, the HPA axis and cortisol, the innate immune system and inflammatory cytokines, ovarian steroids, the GI system, adipose tissue and related peptides, the microbiome, vascular endothelial growth factor, and gene polymorphisms (Duman et al., 2016). These systems influence one’s susceptibility to depression and lead to an increased incidence of depression. Interactions between these systems and the CNS, and within these systems constitute an immense network across organ systems in the pathophysiology of depression. Current efforts are focusing on the identification of specific interactions between these systems, and a large-scale, integrated network or database including all the interactions in the pathophysiology of depression still remains to be generated. Once we attain such a network, we will be able to elevate our study from the molecular level to the systems level, which will revolutionize our understanding of complex biological regulatory interactions in the pathophysiology of depression, as well as providing much needed knowledge to develop new approaches to prevent depression.

Traditional Chinese Medicine is a systems medicine which can play a role in the prevention of depression. Here, we provide an example to further illustrate that TCM could prevent depression at its early stages. Immune dysregulation, specifically of inflammatory processes, is associated with symptoms of depression. In particular, increased levels of circulating pro-inflammatory cytokines and the subsequent activation of brain-resident microglia contribute to neurobiological changes that underlie depression which then lead to depressive behavioral symptoms (Wohleb et al., 2016b). Thus, agents that exert anti-inflammatory activity could be used to prevent depression before immune dysregulation triggers neurobiological changes in the brain. We have recently investigated anti-inflammatory activity of one major category, “Qing-Re-Yao” (medicines that treats inflammation related syndrome) (Guan et al., 2018). A total of 54 herbs in this category were studied by examining their effects on six main mechanisms that underlie inflammation. Our results indicated that 93% of the herbs exerted anti-inflammatory activity *via* at least one underlying mechanism and 68% *via* two or more mechanisms. TCM formulas that aim to alleviate inflammation-induced depressive symptoms generally contain at least one herb in this category. Thus, we asked if “Qing-Re-Yao”-containing antidepressant formulas can be used to prevent inflammation-induced depressive symptoms. Several benefits have generally been acknowledged. First, TCM herbs can be used on a daily basis, like most off-counter daily supplements in pharmacy stores. Second, most TCM herbs exert their action *via* multiple mechanisms simultaneously. Compared to single-target drugs, multi-targeted drugs have more potential to treat an illness with multiple symptoms, such as depression. Third, TCM is a personalized medicine. Susceptibility to external challenges and resulting biological changes vary from person to person. Inflammation can be caused by different underlying mechanisms and corresponding treatments need to be adjusted to fit each individual. Altogether, “Qing-Re-Yao” is a good candidate for preventative anti-inflammation medicine and could play an irreplaceable role in the prevention of inflammation-associated depressive symptoms.

In summary, for TCM, both challenges and opportunities exist in the new era of antidepressant discovery. The clinical experience and “systems” concept of TCM for diagnosis and treatment, could provide an alternative way to improve our understanding of the pathophysiological mechanisms that underlie depression as well as provide ways to discover novel antidepressant agents or approaches. We believe that translational medicine for complex human diseases, such as depression, would benefit from the combination of principle modern molecular medicine with certain ideas of empirical TCM.

## Author Contributions

All authors listed have made a substantial, direct and intellectual contribution to the work, and approved it for publication.

## Conflict of Interest Statement

The authors declare that the research was conducted in the absence of any commercial or financial relationships that could be construed as a potential conflict of interest.

## References

[B1] Abdallah C. G. Sanacora G. Duman R. S. Krystal J. H. (2015). Ketamine and rapid-acting antidepressants: a window into a new neurobiology for mood disorder therapeutics. *Annu. Rev. Med.* 66 509–523. 10.1146/annurev-med-053013-062946 25341010 PMC4428310

[B2] Ahokas A. Kaukoranta J. Wahlbeck K. Aito M. (2001). Estrogen deficiency in severe postpartum depression: successful treatment with sublingual physiologic 17 beta-estradiol: a preliminary study. *J. Clin. Psychiatry* 62 332–336. 10.4088/JCP.v62n0504 11411813

[B3] Arseneault-Breard J. Rondeau I. Gilbert K. Girard S. A. Tompkins T. A. Godbout R. (2012). Combination of *Lactobacillus helveticus* R0052 and *Bifidobacterium longum* R0175 reduces post-myocardial infarction depression symptoms restores intestinal permeability in a rat model. *Br. J. Nutr.* 107 1793–1799. 10.1017/S0007114511005137 21933458

[B4] Bangasser D. A. Valentino R. J. (2014). Sex differences in stress-related psychiatric disorders: neurobiological perspectives. *Front. Neuroendocrinol.* 35:303–319. 10.1016/j.yfrne.2014.03.008 24726661 PMC4087049

[B5] Berman R. M. Cappiello A. Anand A. Da O. Heninger G. R. Charney D. S. (2000). Antidepressant effects of ketamine in depressed patients. *Soc. Biol. Psychiatry* 47 351–354. 10.1016/S0006-3223(99)00230-9 10686270

[B6] Berton O. Nestler E. J. (2006). New approaches to antidepressant drug discovery: beyond monoamines. *Nat. Rev.* 7 137–151. 10.1038/nrn1846 16429123

[B7] Bravo J. A. Forsythe P. Chew M. V. Escaravage E. Savignac H. M. Dinan T. G. (2011). Ingestion of *Lactobacillus* strain regulates emotional behavior and central GABA receptor expression in a mouse via the vagus nerve. *Proc. Natl. Acad. Sci. U.S.A.* 108 16050–16055. 10.1073/pnas.1102999108 21876150 PMC3179073

[B8] Bulling S. Schicker K. Zhang Y. W. Steinkellner T. Stockner T. Gruber C. W. (2012). The mechanistic basis for noncompetitive ibogaine inhibition of serotonin and dopamine transporters. *J. Biol. Chem.* 287 18524–18534. 10.1074/jbc.M112.343681 22451652 PMC3365767

[B9] Burgdorf J. Zhang X. Nicholson K. L. Balster R. L. Leander J. D. Stanton P. K. (2013). GLYX-13, a NMDA receptor glycine-site functional partial agonist, induces antidepressant-like effects without ketamine-like side effects. *Neuropsychopharmacology* 38 729–742. 10.1038/npp.2012.246 23303054 PMC3671991

[B10] Butcher E. C. (2005). Can cell systems biology rescue drug discovery? *Nat. Rev.* 4 461–467. 10.1038/nrd1754 15915152

[B11] Charney D. S. (2004). Psychobiological mechanisms of resilience and vulnerability: implications for successful adaptation to extreme stress. *Am. J. Psychiatry* 161 195–216. 10.1176/appi.ajp.161.2.195 14754765

[B12] Desbonnet L. Garrett L. Clarke G. Bienenstock J. Dinan T. G. (2008). The probiotic *Bifidobacteria infantis*: an assessment of potential antidepressant properties in the rat. *J. Psychiatr. Res.* 43 164–174. 10.1016/j.jpsychires.2008.03.009 18456279

[B13] Diaz Heijtz R. Wang S. Anuar F. Qian Y. Björkholm B. Samuelsson A. (2011). Normal gut microbiota modulates brain development and behavior. *Proc. Natl. Acad. Sci. U.S.A.* 108 3047–3052. 10.1073/pnas.1010529108 21282636 PMC3041077

[B14] Drevets W. C. Zarate C. A. Furey M. L. (2013). Antidepressant effects of the muscarinic cholinergic receptor antagonist scopolamine: a review. *Biol. Psychiatry* 73 1156–1163. 10.1016/j.biopsych.2012.09.031 23200525 PMC4131859

[B15] Duman R. S. (2017). Sex-specific disease-associated modules for depression. *Nat. Med.* 23 1015–1017. 10.1038/nm.4391 28886002 PMC5813287

[B16] Duman R. S. (2018). Ketamine and rapid-acting antidepressants: a new era in the battle against depression and suicide. *F1000Res* 7:F1000 Faculty Rev-659. 10.12688/f1000research.14344.1 29899972 PMC5968361

[B17] Duman R. S. Aghajanian G. K. Sanacora G. Krystal J. H. (2016). Synaptic plasticity and depression: new insights from stress and rapid-acting antidepressants. *Nat. Med.* 22 238–249. 10.1038/nm.4050 26937618 PMC5405628

[B18] Feng D. D. Tang T. Lin X. P. Yang Z. Y. Yang S. Xia Z. A. (2016). Nine traditional Chinese herbal formulas for the treatment of depression: an ethnopharmacology, phytochemistry, and pharmacology review. *Neuropsychiatr. Dis. Treat.* 12 2387–2402. 10.2147/NDT.S114560 27703356 PMC5036551

[B19] Gerhard D. M. Duman R. S. (2018). Rapid-acting antidepressants: mechanistic insights and future directions. *Curr. Behav. Neurosci. Rep.* 5 36–47. 10.1007/s40473-018-0139-8 30034992 PMC6051539

[B20] Guan F. Lam W. Hu R. Kim Y. K. Han H. Cheng Y. C. (2018). Majority of Chinese medicine herb category “Qing Re Yao” have multiple mechanisms of anti-inflammatory activity. *Sci. Rep.* 8:7416. 10.1038/s41598-018-25813-x 29743639 PMC5943244

[B21] Hasebe T. Ueno N. Musch M. W. Nadimpalli A. Kaneko A. Kaifuchi N. (2016). Daikenchuto (TU-100) shapes gut microbiota architecture and increases the production of ginsenoside metabolite compound K. *Pharmacol. Res. Perspect.* 4:e00215. 10.1002/prp2.215 26977303 PMC4777267

[B22] Jacobs M. Zhang Y. W. Campbell S. D. Rudnick G. (2007). Ibogaine, a noncompetitive inhibitor of serotonin transport, acts by stabilizing the cytoplasmic-facing form of the transporter. *J. Biol. Chem.* 282 29441–29447. 10.1074/jbc.M704456200 17698848

[B23] Jin X. L. Shibata C. Naito H. Ueno T. Funayama Y. Fukushima K. (2001). Intraduodenal and intrajejunal administration of the herbal medicine, dai-kenchu-tou, stimulates small intestinal motility via cholinergic receptors in conscious dogs. *Dig. Dis. Sci.* 46 1171–1176. 10.1023/A:1010690624187 11414290

[B24] Jin Z. L. Gao N. Li X. R. Tang Y. Xiong J. Chen H. X. (2015). The antidepressant-like pharmacological profile of Yuanzhi-1, a novel serotonin, norepinephrine and dopamine reuptake inhibitor. *Eur. Neuropsychopharmacol.* 25 544–556. 10.1016/j.euroneuro.2015.01.005 25638027

[B25] Jin Z. L. Gao N. Zhang J. R. Li X. R. Chen H. X. Xiong J. (2014). The discovery of Yuanzhi-1, a triterpenoid saponin derived from the traditional Chinese medicine, has antidepressant-like activity. *Prog. Neuropsychopharmacol. Biol. Psychiatry* 53 9–14. 10.1016/j.pnpbp.2014.02.013 24614095

[B26] Jing L. L. Zhu X. X. Lv Z. P. Sun X. G. (2015). Effect of *Xiaoyaosan* on major depressive disorder. *Chin. Med.* 10:18. 10.1186/s13020-015-0050-0 26191079 PMC4506593

[B27] Kuehner C. (2017). Why is depression more common among women than among men? *Lancet Psychiatry* 4 146–158. 10.1016/S2215-0366(16)30263-2 27856392

[B28] Labonté B. Engmann O. Purushothaman I. Menard C. Wang J. Tan C. (2017). Sex-specific transcriptional signatures in human depression. *Nat. Med.* 23 1102–1111. 10.1038/nm.4386 28825715 PMC5734943

[B29] Lepack A. E. Fuchikami M. Dwyer J. M. Banasr M. Duman R. S. (2015). BDNF release is required for the behavioral actions of ketamine. *Int. J. Neuropsychopharmacol.* 18:pyu033. 10.1093/ijnp/pyu033 25539510 PMC4368871

[B30] Li N. Liu R. J. Dwyer J. M. Banasr M. Lee B. Son H. (2011). Glutamate N-methyl-D-aspartate receptor antagonists rapidly reverse behavioral and synaptic deficits caused by chronic stress exposure. *Biol. Psychiatry* 69 754–761. 10.1016/j.biopsych.2010.12.015 21292242 PMC3068225

[B31] Li Y. Chen Z. Yu N. Yao K. Che Y. Xi Y. (2016). Chinese herbal medicine for postpartum depression: a systematic review of randomized controlled trials. *Evid. Based Complement. Alternat. Med.* 2016:5284234. 10.1155/2016/5284234 27774110 PMC5059536

[B32] Maeng S. Zarate C. A. Du J. Schloesser R. J. McCammon J. Chen G. (2008). Cellular mechanisms underlying the antidepressant effects of ketamine: role of alpha-amino-3-hydroxy-5-methylisoxazole-4-propionic acid receptors. *Biol. Psychiatry* 63 349–352. 10.1016/j.biopsych.2007.05.028 17643398

[B33] Manji H. K. Drevets W. C. Charney D. S. (2001). The cellular neurobiology of depression. *Nat. Med.* 7 541–547. 10.1038/87865 11329053

[B34] Mazure C. M. Jones D. P. (2015). Twenty years and still counting: including women as participants and studying sex and gender in biomedical research. *BMC Womens Health* 15:94. 10.1186/s12905-015-0251-9 26503700 PMC4624369

[B35] Murata P. Kase Y. Ishige A. Sasaki H. Kurosawa S. Nakamura T. (2002). The herbal medicine Dai-kenchu-to and one of its active components [6]-shogaol increase intestinal blood flow in rats. *Life Sci.* 70 2061–2070. 10.1016/S0024-3205(01)01552-1 12148698

[B36] Nestler E. J. Barrot M. DiLeone R. J. Eisch A. J. Gold S. J. Monteggia L. M. (2000). Neurobiology of depression. *Neuron* 34 13–25. 10.1016/S0896-6273(02)00653-0 11931738

[B37] Parker G. B. Brotchie H. L. (2004). From diathesis to dimorphism: the biology of gender differences in depression. *J. Nerv. Ment. Dis.* 192 210–216. 10.1097/01.nmd.0000116464.60500.63 15091302

[B38] Preskorn S. H. Baker B. Kolluri S. Menniti F. S. Krams M. Landen J. W. (2008). An innovative design to establish proof of concept of the antidepressant effects of the NR2B subunit selective N-methyl-D-aspartate antagonist, CP-101,606, in patients with treatment-refractory major depressive disorder. *J. Clin. Psychopharmacol.* 28 631–637. 10.1097/JCP.0b013e31818a6cea 19011431

[B39] Rhee S. Pothoulakis C. Mayer E. A. (2009). Principles and clinical implications of the brain-gut-enteric microbiota axis. *Nat. Rev. Gastroenterol. Hepatol.* 6 306–314. 10.1038/nrgastro.2009.35 19404271 PMC3817714

[B40] Schroeder F. A. Lin C. L. Crusio W. E. Akbarian S. (2007). Antidepressant-like effects of the histone deacetylase inhibitor, sodium butyrate, in the mouse. *Biol. Psychiatry* 62 55–64. 10.1016/j.biopsych.2006.06.036 16945350

[B41] Soldin O. P. Mattison D. R. (2009). Sex differences in pharmacokinetics and pharmacodynamics. *Clin. Pharmacokinet.* 48 143–157. 10.2165/00003088-200948030-00001 19385708 PMC3644551

[B42] Tokita Y. Satoh K. Sakaguchi M. Endoh Y. Mori I. Yuzurihara M. (2007). The preventive effect of Daikenchuto on postoperative adhesion-induced intestinal obstruction in rats. *Inflammopharmacology* 15 65–66. 10.1007/s10787-006-1552-2 17450444

[B43] Ung C. Y. Li H. Cao Z. W. Li Y. X. Chen Y. Z. (2007). Are herb-pairs of traditional Chinese medicine distinguishable from others? Pattern analysis and artificial intelligence classification study of traditionally defined herbal properties. *J. Ethnopharmacol.* 111 371–377. 10.1016/j.jep.2006.11.037 17267151

[B44] Voleti B. Navarria A. Liu R. J. Banasr M. Li N. Terwilliger R. (2013). Scopolamine rapidly increases mammalian target of rapamycin complex 1 signaling, synaptogenesis, and antidepressant behavioral responses. *Biol. Psychiatry* 74 742–749. 10.1016/j.biopsych.2013.04.025 23751205 PMC3773272

[B45] Wohleb E. S. Franklin T. Iwata M. Duman R. S. (2016a). Integrating neuroimmune systems in the neurobiology of depression. *Nat. Rev. Neurosci.* 17 497–511. 10.1038/nrn.2016.69 27277867

[B46] Wohleb E. S. Wu M. Gerhard D. M. Taylor S. R. Picciotto M. R. Alreja M. (2016b). GABA interneurons mediate the rapid antidepressant-like effects of scopolamine. *J. Clin. Invest.* 126 2482–2494. 10.1172/JCI85033 27270172 PMC4922686

[B47] Wu Y. C. Hsieh C. L. (2011). Pharmacological effects of *Radix Angelica Sinensis (Danggui)* on cerebral infarction. *Chin. Med.* 6:32. 10.1186/1749-8546-6-32 21867503 PMC3174116

[B48] Yang L. Di Y. M. Shergis J. L. Li Y. Zhang A. L. Lu C. (2018). A systematic review of acupuncture and Chinese herbal medicine for postpartum depression. *Complement. Ther. Clin. Pract.* 33 85–92. 10.1016/j.ctcp.2018.08.006 30396632

[B49] Yeung W. F. Chung K. F. Ng K. Y. Yu Y. M. Zhang S. P. Ng B. F. (2015). Prescription of Chinese herbal medicine in pattern-based traditional chinese medicine treatment for depression: a systematic review. *Evid. Based Complement. Alternat. Med.* 2015:160189. 10.1155/2015/160189 26180532 PMC4477207

[B50] Yoshikawa K. Shimada M. Kuwahara T. Hirakawa H. Kurita N. Sato H. (2013). Effect of Kampo medicine “Dai-kenchu-to” on microbiome in the intestine of the rats with fast stress. *J. Med. Invest.* 60 221–227. 10.2152/jmi.60.221 24190039

[B51] Zarate C. A. Singh J. B. Carlson P. J. Brutsche N. E. Ameli R. Luckenbaugh D. A. (2006). A randomized trial of an N-methyl-D-aspartate antagonist in treatment-resistant major depression. *Arch. Gen. Psychiatry* 63 856–864. 10.1001/archpsyc.63.8.856 16894061

[B52] Zhou W. Wang N. Yang C. Li X. M. Zhou Z. Q. Yang J. J. (2014). Ketamine-induced antidepressant effects are associated with AMPA receptors-mediated upregulation of mTOR and BDNF in rat hippocampus and prefrontal cortex. *Eur. Psychiatry* 29 419–423. 10.1016/j.eurpsy.2013.10.005 24321772

